# Arabidopsis ECHIDNA protein is involved in seed coloration, protein trafficking to vacuoles, and vacuolar biogenesis

**DOI:** 10.1093/jxb/eraa147

**Published:** 2020-03-23

**Authors:** Takuji Ichino, Kazuki Maeda, Ikuko Hara-Nishimura, Tomoo Shimada

**Affiliations:** 1 Department of Botany, Graduate School of Science, Kyoto University, Kyoto, Japan; 2 Department of Plant Developmental Biology, Centre for Organismal Studies, Heidelberg University, Heidelberg, Germany; 3 Laboratory of Plant Gene Expression, Research Institute for Sustainable Humanosphere, Kyoto University, Uji, Japan; 4 Department of Biology, Faculty of Science and Engineering, Konan University, Kobe, Japan; 5 University of Maryland, USA

**Keywords:** *Arabidopsis thaliana*, ECHIDNA, GREEN FLUORESCENT SEED 9, mucilage, protein sorting, seed coloration, *trans*-Golgi network, vacuolar morphology, vacuolar trafficking, vacuole

## Abstract

Flavonoids are a major group of plant-specific metabolites that determine flower and seed coloration. In plant cells, flavonoids are synthesized at the cytosolic surface of the endoplasmic reticulum and are sequestered in the vacuole. It is possible that membrane trafficking, including vesicle trafficking and organelle dynamics, contributes to flavonoid transport and accumulation. However, the underlying mechanism has yet to be fully elucidated. Here we show that the Arabidopsis ECHIDNA protein plays a role in flavonoid accumulation in the vacuole and protein trafficking to the vacuole. We found defective pigmentation patterns in *echidna* seed, possibly caused by reduced levels of proanthocyanidins, which determine seed coloration. The *echidna* mutant has defects in protein sorting to the protein storage vacuole as well as vacuole morphology. These findings indicate that ECHIDNA is involved in the vacuolar trafficking pathway as well as the previously described secretory pathway. In addition, we found a genetic interaction between *echidna* and *green fluorescent seed 9* (*gfs9*), a membrane trafficking factor involved in flavonoid accumulation. Our findings suggest that vacuolar trafficking and/or vacuolar development, both of which are collectively regulated by ECHIDNA and GFS9, are required for flavonoid accumulation, resulting in seed coat pigmentation.

## Teaser

An *Arabidopsis thaliana* mutant lacking the *trans*-Golgi network-localized protein ECHIDNA shows vacuole-related phenotypes, including altered seed coloration, vacuolar protein sorting, and vacuolar morphology.

## Introduction

A variety of metabolites unique to plants are produced as a plant strategy to adapt to the surrounding environment. Flavonoids are one group of secondary metabolites that are present in most land plants. Flavonoids act as pigments that determine flower and fruit coloration to attract pollinators and seed dispersers for plant reproduction and propagation (Kevan and Baker, 1983; Grotewold, 2006; Sheehan *et al.*, 2016). Plant seeds accumulate flavonoids in their seed coats. Arabidopsis seed contains two types of flavonoids, flavonols and proanthocyanidins, also known as condensed tannins (Routaboul *et al.*, 2006). These seed flavonoids protect the embryo and endosperm inside the seed from pathogens and UV radiation. When flavonoid accumulation is blocked by the lack of associated molecular components, the seeds are pale tan in color, designated as *transparent testa* (*tt*) based on their mutant phenotype. Using genetic and molecular approaches, researchers have identified the detailed mechanism of flavonoid biosynthesis and its transcriptional regulation system (Lepiniec *et al.*, 2006).

Flavonoid biosynthesis starts in the phenylpropanoid pathway in plant cells. Most of the enzymes required for flavonoid biosynthesis form a protein complex referred to as the flavonoid metabolon and are located on the cytosolic surface of the endoplasmic reticulum (ER) membrane (Lepiniec *et al.*, 2006). Most flavonoids are amassed as pigments in the vacuole, where they are destined to be polymerized and chemically decorated by glycosylation, methylation, and acylation. The transport of flavonoids has been shown to involve membrane transporters located in endomembrane compartments. In *Arabidopsis thaliana*, the multidrug and toxic compound extrusion (MATE) family protein TT12 localizes on the vacuolar membrane of seed coat cells and functions in the uptake of flavonoid monomers into the vacuole (Debeaujon *et al.*, 2001; Marinova *et al.*, 2007). There are several orthologues of TT12 in *Vitis vinifera* and *Medicago truncatula* with flavonoid transport activity (Gomez *et al.*, 2009; Zhao and Dixon, 2009). The ATP-binding cassette proteins in *Zea mays*, *Glycine max*, and *V. vinifera* function in the transport of flavonoid derivatives across the membrane (Goodman *et al.*, 2004; Sugiyama *et al.*, 2007; Francisco *et al.*, 2013).

In addition to a transporter-mediated flavonoid transport system, vesicle-mediated trafficking is involved in flavonoid accumulation. In the tapetum cells of *Brassica* anthers, an ER-derived organelle, the tapetosome, contains flavonoids and lipids that are delivered to the pollen surface (Hsieh and Huang, 2007). The transport of anthocyanin, a pigmented flavonoid, to the vacuole in Arabidopsis leaf cells is affected by treatment with brefeldin A, an inhibitor of the vesicle trafficking machinery used for protein sorting (Poustka *et al.*, 2007). More recent observations have suggested that the machinery for autophagy, an intracellular recycling system, affects the vacuolar accumulation of anthocyanins (Kulich *et al.*, 2013). However, it remains controversial whether the autophagy-related components, which participate in the machinery for microautophagy, actually function in anthocyanin trafficking (Pourcel *et al.*, 2010; Chanoca *et al.*, 2015).

We have previously isolated the Arabidopsis mutant *green fluorescent seed 9* (*gfs9*) through forward genetic screening for protein trafficking to the vacuole (Fuji *et al.*, 2007). *gfs9* has a defect in seed coat pigmentation resulting from reduced accumulation of proanthocyanidins and flavonols in the seed (Ichino *et al.*, 2014). Identification of the gene responsible for *gfs9* revealed that *GFS9* is allelic to *TT9* (Shirley *et al.*, 1995; Ichino *et al.*, 2014). GFS9 protein is distributed on the Golgi apparatus and functions in the trafficking of vacuolar proteins and in vacuolar development (Ichino *et al.*, 2014). Although analysis of GFS9 has identified the role of the membrane trafficking machinery in flavonoid accumulation, the detailed mechanism linking membrane trafficking to flavonoid accumulation still is unknown. The question remains whether membrane trafficking factors other than GFS9 are involved in flavonoid accumulation. It is also unclear whether any other endomembrane compartments except for the Golgi apparatus affect flavonoid accumulation.

In this study, we identified another Arabidopsis trafficking mutant, *echidna*, which resulted in defective seed coloration, possibly as a result of reduced accumulation of proanthocyanidins. The ECHIDNA protein localizes to the *trans*-Golgi network (TGN) (Gendre *et al.*, 2011; Drakakaki *et al.*, 2012) and functions in secretory pathways, including the secretion of soluble apoplast proteins (Gendre *et al.*, 2011) and membrane proteins located on the plasma membrane, such as auxin carriers (e.g. AUX1, PIN3) (Boutte *et al*., 2013). We found that a lack of *ECHIDNA* resulted in two kinds of membrane trafficking defects, in vacuolar protein sorting and vacuolar morphology. We also found an enhanced effect on plant growth with the combination of the *echidna* and *gfs9* mutations. These findings demonstrate that TGN-localized ECHIDNA and Golgi-localized GFS9 orchestrate the intracellular trafficking of proteins and flavonoids, resulting in the regulation of plant development and seed coloration.

## Materials and methods

### Plant materials and growth conditions

We used *Arabidopsis thaliana* accessions Columbia-0 (CS60000) and Columbia-3 as the wild-type plants in this study. We used two lines of Arabidopsis T-DNA insertion mutants, *echidna* (SAIL_163_E09) and *gfs9-3* (SALK_057766, described as *gfs9* throughout this manuscript), which have been previously reported (Gendre *et al.*, 2011; Ichino *et al.*, 2014). The Arabidopsis seeds were surface-sterilized with 70% ethanol and then sown on to either 0.5% (w/v) gellan gum (Wako, Osaka, Japan) or 0.8% (w/v) agar containing 1% (w/v) sucrose and Murashige and Skoog medium (Wako). The seeds were incubated at 4 °C for 2 days to break seed dormancy. We grew the Arabidopsis plants side by side on plates at 22 °C for approximately 1 month under continuous light (100 µmol s^−1^ m^−2^). Then, we transferred the plants on to vermiculite or peat moss (Sakata seed, Yokohama, Japan) for subsequent growth.

We crossed the *echidna* mutant with the *gfs9* mutant to generate the *echidna gfs9* double mutant. The F_2_ plants were grown on a medium containing 10 mg l^−1^ glufosinate ammonium (Sigma-Aldrich, St. Louis, USA) and 50 mg l^−1^ kanamycin (Sigma-Aldrich) to select the plant lines bearing the T-DNA insertions in both the *ECHIDNA* and *GFS9* genes. After identifying the F_2_ plants with the homozygous *gfs9* allele and the heterozygous *echidna* allele, we selected the *echidna gfs9* double mutant from the progeny of F_2_ self-fertilization based on the *echidna* dwarf phenotype. The following Arabidopsis transgenic lines were also used: SP-GFP-CT24 (Fuji *et al.*, 2007), mCherry-VAMP711, and mCherry-ARA7 (Geldner *et al.*, 2009). We crossed the transgenic lines with the *echidna* mutant as described above to examine these proteins in the *echidna* mutant cell.

### Genotyping

We identified T-DNA by PCR-based genotyping with genomic DNA extracted from the seedling leaves. We confirmed T-DNA insertion in the *ECHIDNA* gene (the *echidna* allele) by PCR using a combination of the primers LB1 (5′-AAA AATGAAGTTGTTTAAAGTAGGTA-3′) and SAIL_163_E09-RP (5′-AGAGAAGAGTTATCGGGCTCG-3′). The wild-type allele in the *ECHIDNA* gene was confirmed by PCR using a combination of the primers SAIL_163_E09-LP (5′-AAACGGAAAGGGAAACACAAC-3′) and SAIL_163_E09-RP. We confirmed the T-DNA insertion in the *GFS9* gene (the *gfs9* allele) by PCR using a combination of the primers LBa1 (5′-TGGTTCACGTAGTGGGCCATCG-3′) and At3g28430-4-R (5′-ATAGGCGGCTGCTCGGGTAA-3′). Finally, the wild-type allele in *GFS9* was confirmed by PCR using a combination of the primers At3g28430-4-F (5′-GCCAGGGAAAGTCTCATCTGC-3′) and At3g28430-4-R.

### SDS-PAGE and immunoblot analysis

SDS-PAGE and immunoblot analysis were performed as described previously (Shimada *et al.*, 2003a, b). The dry seeds were homogenized with a pellet mixer (Treff, Degersheim, Switzerland) in 1× SDS sample buffer [50 mM Tris–HCl (pH 6.8), 1% (w/v) SDS, 5% 2-mercaptoethanol, 10% glycerol, 0.1% bromophenol blue]. The homogenates were heat-treated at 99 °C for 5 min. After centrifugation, the supernatant was analyzed using SDS-PAGE.

We used an anti-12S globulin antibody (diluted 10 000-fold) (Shimada *et al.*, 2003b) for immunoblotting. Signals were detected with an enhanced chemiluminescence detection system (GE Healthcare, Cambridge, UK) and LAS-3000 (Fujifilm, Tokyo, Japan).

### Staining with *p*-dimethylaminocinnamaldehyde, propidium iodide, and india ink

To detect the accumulation of proanthocyanidin and its precursors, Arabidopsis seeds were stained with *p*-dimethylaminocinnamaldehyde as described in previous studies (Abrahams *et al.*, 2002; Baxter *et al.*, 2005). The dry seeds were stained with 2% (w/v) *p*-dimethylaminocinnamaldehyde (Tokyo Chemical Industry, Tokyo, Japan), 50% (w/v) methanol, and 3 M HCl for 1 week. Then, the stained seeds were washed four times in 70% (v/v) ethanol.

We used the fluorescent agent propidium iodide to detect root cap mucilage, as described previously (Rounds *et al.*, 2011; Maeda *et al.*, 2019). Seedlings 9 days after sowing were stained with 10 µg ml^−1^ propidium iodide (Nacalai Tesque, Kyoto, Japan) for ~5 min and then mounted on a glass slide. The root caps were visualized under a confocal microscope.

The amount of root cap mucilage was quantified by staining with india ink, as described previously (Hawes *et al.*, 2000; Maeda *et al.*, 2019). We excised root tips (<1 cm) from vertically grown seedlings 9 days after sowing and placed them gently in 100 µl of 50% (v/v) india ink (Trusco Nakayama, Tokyo, Japan) on a glass slide using a toothpick. The unstained mucilage was observed using a light microscope (Axioskop 2 plus, Carl Zeiss, Jena, Germany) with a cover slip (24×60; Matsunami, Osaka, Japan) on the glass slide. We acquired images and segmented the images into regions representing mucilage or various tissues with ImageJ (http://rsb.info.nih.gov/ij) and its plugin, Trainable Weka Segmentation (Arganda-Carreras *et al.*, 2017).

### Histological analysis

Root tips obtained from seedlings on day 9 after sowing were fixed with 4% (w/v) paraformaldehyde and 1% (v/v) glutaraldehyde in 50 mM cacodylate buffer (pH 7.4) for 2 hours. We followed the procedures described by Nishimura *et al.* (1993). The samples were dehydrated in a graded ethanol series at room temperature and embedded in LR White resin (London Resin, Basingstoke, UK). The blocks were polymerized under a UV lamp at −20 °C for 24 hours. We stained thin sections with toluidine blue and examined the sections with an Axioskop 2 plus light microscope.

### Light microscopy

Arabidopsis seeds were observed by bright-field microscopy with a stereo microscope (SZX12; Olympus, Tokyo, Japan) equipped with a CCD camera (DP20; Olympus). To facilitate comparison of seed coloration, all images were acquired using the same parameters on the microscope and camera.

### Fluorescent microscopy

We used a research Macro Zoom Fluorescence Microscope (MVX10; Olympus) equipped with a CCD camera (VB-7000; Keyence, Osaka, Japan) to detect the green fluorescent protein (GFP) fluorescence in dry seeds expressing SP-GFP-CT24. We acquired all images using the same parameters on the microscope and camera to facilitate comparison of the intensity of fluorescence.

### Confocal laser scanning microscopy

We removed the seed coat and endosperm from the seeds in glycerol to pick out the internal embryo in preparation to observe the GFP fluorescence of SP-GFP-CT24 and autofluorescence of protein storage vacuoles (PSVs). Seedlings were mounted in water on a microscope slide to prevent plasmolysis and examined using confocal laser scanning microscopy.

We used an LSM780 confocal laser scanning microscope (Carl Zeiss) to detect SP-GFP-CT24 fluorescence [excitation 488 nm (argon laser), emission 491–550 nm], mCherry-VAMP711 fluorescence, mCherry-ARA7 fluorescence, autofluorescence of PSVs [excitation 561 nm (diode-pumped solid-state laser), emission 560–656 nm], and the fluorescence of propidium iodide [excitation 561 nm (diode-pumped solid-state laser), emission 578–663 nm]. We captured images using a 63×1.20 numerical aperture water immersion objective (C-Apochromat, 441777-9970-000; Carl Zeiss).

We used another confocal laser scanning microscope (Leica TCS SP5 II, Leica Microsystems, Wetzlar, Germany) with a ×63.0 water immersion objective (HCX PL APO lambda blue 63.0×1.20 WATER UV; Leica) to visualize mCherry-ARA7 fluorescence [excitation 561 nm (DPSS laser), emission 615–676 nm] of the root cells. The acquired images were processed and analyzed using ZEN 2009 Light Edition, ZEN2011 Light Edition (Carl Zeiss), LAS AF Lite (Leica Microsystems), Adobe Photoshop Elements 9 (Adobe Systems, San Jose, USA), and ImageJ.

### Statistical analysis

We analyzed multiple comparisons of seedling root length, plant height, and plant diameter using Tukey’s honestly significant difference (HSD) test (α=0.01) using the R 3.3.2 package (R Core Team, 2016). Multiple comparisons of PSV diameter and the signal intensity of mCherry-ARA7 were also analyzed by Tukey’s HSD test (α=0.05). Comparisons of the number and size of ARA7-positive endosomes, the ratio of signal intensity, deviation of PSV size, and area devoid of india ink were performed with Student’s *t*-test.

### Accession numbers

Arabidopsis Genome Initiative locus identifiers for the genes mentioned in this study are as follows: ECHIDNA, At1g09330; GFS9, At3g28430; VAMP711, At4g32150; ARA7, At4g19640.

## Results

### ECHIDNA is involved in seed coloration

**Fig. 1.**
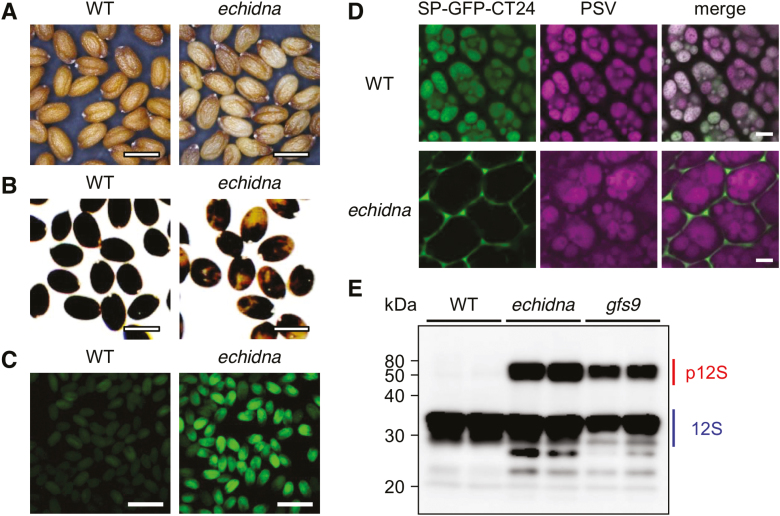
Seed color phenotype and missorting of vacuolar proteins in *echidna* mutant seeds. (A) Seeds of wild-type (WT) and *echidna* mutant. (B) Seeds of WT and *echidna* mutant after staining with *p*-dimethylaminocinnamaldehyde. (C) GFP fluorescence images of WT and *echidna* dry seeds expressing vacuolar-targeted GFP, SP-GFP-CT24. (D) Confocal microscopic images of vacuolar-targeted GFP in embryonic cotyledon cells of WT and *echidna* dry seeds. Vacuolar-targeted GFP fluorescent signal and autofluorescence of the protein storage vacuole (PSV) are shown. (E) Immunoblot analysis of seed storage protein in WT, *echidna*, and *gfs9* seeds. Antibody to 12S globulin was used. The *gfs9* mutant is known to be defective in protein sorting to the vacuole. p12S, precursor forms of 12S globulin; 12S, mature form of 12S globulin. Bars=0.5 mm (A, B), 1 mm (C), and 5 μm (D).

Previously, we reported a molecular link between membrane trafficking and flavonoid accumulation in the vacuole (Ichino *et al.*, 2014). Further investigation revealed that the Arabidopsis trafficking mutant *echidna* produced grayish-tan seeds (Fig. 1A). Microscopic observation showed that *echidna* seeds exhibit a pale brown color with a partially whitish seed body (Fig. 1A). The wild-type seeds displayed a dark brown color throughout the whole seed body (Fig. 1A).

The coloration of plant seeds is determined by the quantity and quality of accumulated flavonoids. We stained the *echidna* seeds with *p*-dimethylaminocinnamaldehyde to detect the seed flavonoid proanthocyanidin. This reagent easily reacts with proanthocyanidins and its precursors to stain Arabidopsis seeds black (Abrahams *et al.*, 2002). After treatment with *p*-dimethylaminocinnamaldehyde, the wild-type seeds were completely stained black, whereas the *echidna* seeds were partially stained black (Fig. 1B). Some regions of *echidna* seed bodies were a tan color (Fig. 1B). These results suggest that ECHIDNA protein is required for seed coloration, possibly via a mechanism involving proanthocyanidin accumulation in the seeds.

### ECHIDNA is involved in vacuolar protein sorting in seeds

ECHIDNA protein, which localizes to the TGN, has been shown to function in a secretory pathway to transport proteins and cell wall components, including polysaccharides, mucilage, and wax (Gendre et al., 2011, 2013; McFarlane et al., 2013, 2014). Currently, the involvement of ECHIDNA in vacuolar trafficking is unclear. Therefore, we investigated vacuolar protein sorting in *echidna* mutant seeds by introducing a vacuolar-targeted GFP, SP-GFP-CT24, which can trace the trafficking route to the PSV in seeds (Nishizawa *et al.*, 2003; Fuji *et al.*, 2007). *echidna* mutant seeds expressing *SP-GFP-CT24* displayed strong GFP fluorescence, whereas wild-type seeds expressing *SP-GFP-CT24* did not show any GFP fluorescence (Fig. 1C). This implies that SP-GFP-CT24 was missorted in the *echidna* mutant seeds because the missorting of SP-GFP-CT24 protein causes aberrant accumulation of this protein in the extracellular space and produces strong GFP fluorescence in seeds (Fuji *et al.*, 2007). In fact, SP-GFP-CT24 was abnormally secreted to outside the cells in *echidna* seed cells (Fig. 1D). In contrast, SP-GFP-CT24 accumulated in PSVs in wild-type seed cells (Fig. 1D). These results suggest that vacuolar protein sorting to the PSV is compromised in the *echidna* mutant.

We performed an immunoblot analysis with anti-12S globulin antibody to identify defects of vacuolar sorting of endogenous proteins in *echidna* mutant seeds. 12S globulin is one of the major seed storage proteins; it is synthesized in the ER in its precursor form and then transported into the PSV, where it is converted into a mature form (Shimada *et al.*, 2003b). We predicted that defects in protein sorting in the vacuolar trafficking pathway would cause abnormal accumulation of the precursor form in dry seeds of *echidna* (Shimada *et al.*, 2003a). In wild-type seeds, 12S globulin appeared only in its mature form (Fig. 1E). In contrast, both the precursor form and the mature form of 12S globulin accumulated abnormally in the *echidna* mutant seeds; a similar pattern of accumulation was observed in *gfs9* mutant seeds (Fig. 1E). These findings confirm that ECHIDNA is involved in the vacuolar trafficking pathway in dry seeds.

We next examined the GFP fluorescence of SP-GFP-CT24 in Arabidopsis seedlings to determine whether ECHIDNA is involved in vacuolar protein transport in general or only in seeds. GFP fluorescence of SP-GFP-CT24 was rarely detected in the hypocotyl and root of both wild-type and *echidna* seedlings on the second day after germination (see Supplementary Fig. S1 at *JXB* online). This was probably due to the low activity of the promoter of the α′-subunit of β-conglycinin, a soybean seed storage protein, to express *SP-GFP-CT24* in these tissues of transgenic Arabidopsis (Nishizawa *et al.*, 2003). This promoter is known to express the *SP-GFP-CT24* gene in seeds but not in leaves of transgenic petunia plants (Beachy *et al.*, 1985). Therefore, we cannot conclude that ECHIDNA is involved in vacuolar protein transport in seedlings.

### ECHIDNA is involved in the proper development of lytic vacuoles and protein storage vacuoles

**Fig. 2.**
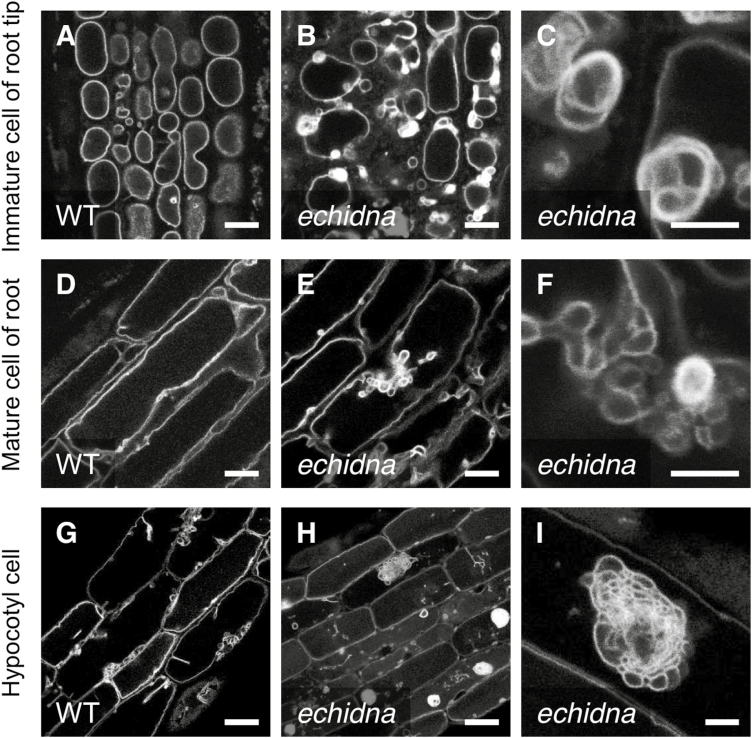
Distribution of vacuolar membrane protein VAMP711 in the *echidna* mutant. Confocal microscopic images of the vacuolar membrane marker mCherry-VAMP711 in wild-type (WT) (A, D, G) and *echidna* mutant (B, C, E, F, H, I) cells. (A–C) Immature epidermal cells of root tip; (D–F) elongated (mature) epidermal cells of seedling root; (G–I) epidermal cells of hypocotyl. Images C, F, and I are magnified views of abnormal structures in the *echidna* mutant. The images in B and C were observed in different seedlings; images in E and F were observed in different fields of view from the same seedling; images in H and I were observed in different fields of view from the same seedling. Bars=10 μm (A, B, D, E), 5 μm (C, F, I), and 20 μm (G, H).

We observed the vacuolar morphology in *echidna* mutant seedlings to investigate whether ECHIDNA protein has a role in vacuolar development. The fluorescent marker mCherry-fused VESICLE ASSOCIATED MEMBRANE PROTEIN 711 (VAMP711), which labels vacuolar membrane (Geldner *et al.*, 2009), was introduced into the *echidna* mutant. Using confocal microscopy, we found aberrant vacuolar morphology in *echidna* mutant cells (Fig. 2). mCherry-VAMP711 labeled the tonoplast of the central vacuole in root epidermal cells of wild-type seedlings (Fig. 2A, D). In contrast, mCherry-VAMP711 accumulated on aberrant structures in the immature epidermal cells of *echidna* seedling roots, although VAMP711 also labeled central vacuolar membranes in the same cells (Fig. 2B). The aberrant structures present in immature cells of *echidna* roots had a much higher intensity of fluorescence than the central vacuolar membranes (Fig. 2B, C). The central vacuole of mature epidermal cells was expanded in both the wild-type and *echidna* mutant seedling roots (Fig. 2D, E). However, we found that the mature epidermal cells of *echidna* roots had abnormal membrane structures labeled by mCherry-VAMP711, which were not apparent in the wild-type cells (Fig. 2E, F).

The abnormal distribution of VAMP711 in the *echidna* mutant was apparent in both root cells and above-ground hypocotyl cells. mCherry-VAMP711 labeled the tonoplast in wild-type hypocotyl cells (Fig. 2G). In *echidna* hypocotyl cells, mCherry-VAMP711 labeled aberrant structures as well as the tonoplast (Fig. 2H). In the *echidna* hypocotyl, a variety of aberrant structures were labeled by mCherry-VAMP711: multi-layered or multi-membrane structures, aggregations of membrane compartments, and unidentified structures (Fig. 2H, I, and Supplementary Fig. S2). These aberrant structures were usually greater than 10 μm in size. These observations demonstrate that vacuolar membrane forms the aberrant structures and aggregations in *echidna* mutant cells, which suggests that ECHIDNA is involved in the proper development of lytic vacuoles.

To examine the effect of ECHIDNA on the development of PSVs in seeds, we observed PSVs and quantitated their diameters (Supplementary Fig. S3). The largest PSV within *echidna* cells was significantly larger than that of wild-type cells, whereas the sizes of the other smaller PSVs within the same cell were comparable between *echidna* and wild-type cells (Supplementary Fig. S3B). The deviation in PSV size of the *echidna* seeds was larger than that of the wild-type seeds (Supplementary Fig. S3C), indicating that PSVs in *echidna* seed cells are more variable in size, whereas PSVs in wild-type seed cells are more uniformly sized. These results suggest that ECHIDNA is involved in regulating PSV morphology in seeds.

### ECHIDNA does not affect late endosomal morphology

**Fig. 3.**
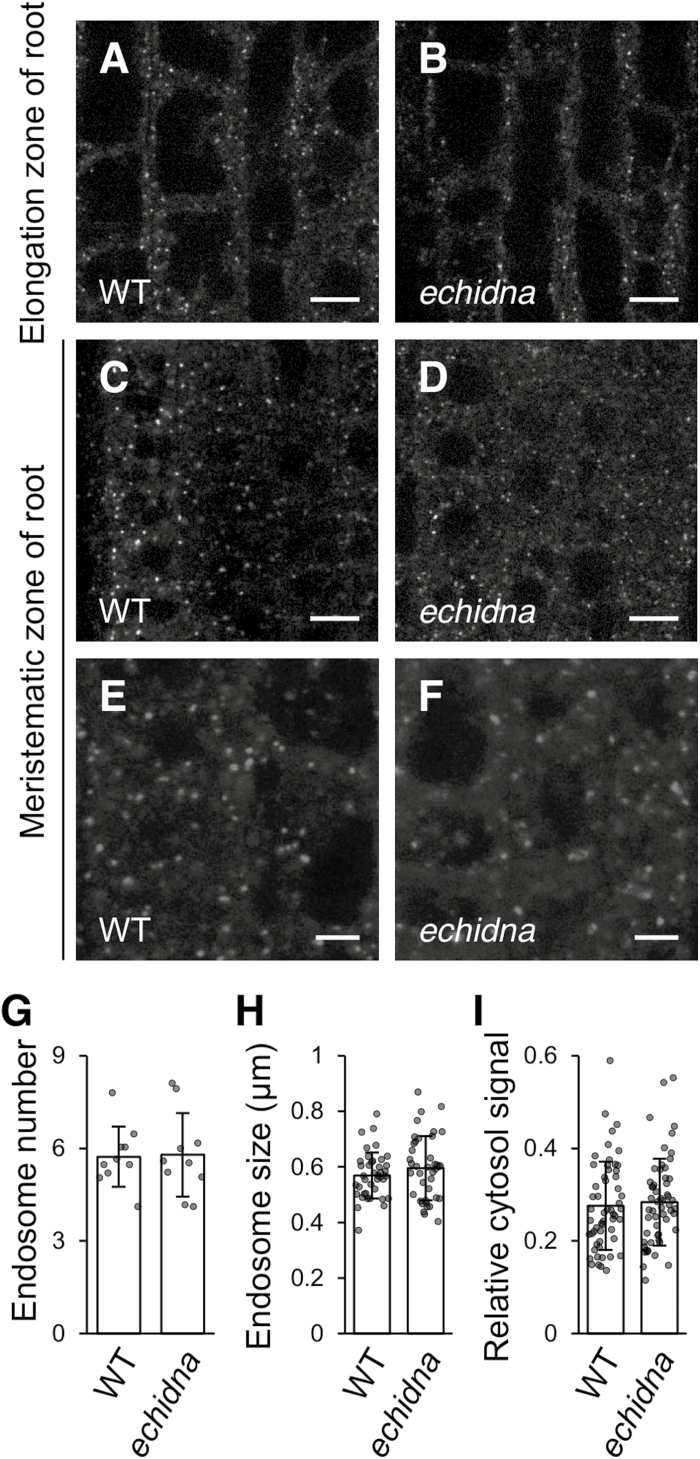
ARA7-labeled late endosomes in the *echidna* mutant. (A–F) Confocal microscopic images of root cells from wild-type (WT) (A, C, E) and *echidna* mutant (B, D, F) seedlings. Late endosomes are labeled with mCherry-ARA7. (A, B) Root cells of the elongation zone; (C–F) root cells of the meristematic zone. Bars=10 μm (A–D) and 5 μm (E, F). (G) Number of ARA7-labeled late endosomes per 100 µm^2^ area in root cells of the elongation zone. Ten plants of each genotype were measured. Each dot represents the mean of three to five regions of interest in a single plant. Each bar graph represents the mean of 10 means ±SD. (H) Size of ARA7-labeled late endosomes in the root cells of the elongation zone. Eight plants of each genotype were measured (five endosomes per plant). Each dot represents the size of a single endosome. Each bar graph represents the mean of 40 endosomes ±SD. (I) Relative signal intensity of cytosol fluorescence, calculated as the ratio of the intensity of cytosolic signal to the intensity of punctate structures within the same cell in the root elongation zone. Fourteen plants of each genotype were measured (four cells per plant). Each dot represents the relative signal intensity of a single cell. Each bar graph represents the mean of 56 cells ±SD. *P* values were calculated using Student’s *t*-test and were as follows: *P*=0.91 (G), *P*=0.24 (H), and *P*=0.66 (I).

The *echidna* mutation causes morphological defects in several endomembrane compartments including the vacuole (Fig. 2), as well as ER and TGN (Gendre *et al.*, 2011; Boutte *et al*., 2013; McFarlane *et al.*, 2014; Ravikumar *et al.*, 2018). Therefore, we investigated whether ECHIDNA has an effect on the morphology of the late endosome. We introduced a fluorescent marker of the late endosome, mCherry-ARA7 (Geldner *et al.*, 2009), into the *echidna* mutant. Using confocal microscopy, we observed ARA7-labeled endosomes as punctate structures in elongated (Fig. 3A, B) and immature (Fig. 3C–F, and Supplementary Fig. S4A) root cells of *echidna* seedlings, similar to the wild-type seedlings. Quantitative analysis revealed no significant differences in the number and size of ARA7-labeled endosomes between wild-type and *echidna* mutant seedlings (Fig. 3G, H). The signal intensity of cytosolic fluorescence and relative intensities of cytosolic fluorescence compared with endosome fluorescence were similar between wild-type and *echidna* mutant seedlings (Fig. 3I and Supplementary Fig. S4B). These results suggest that ECHIDNA protein has little or no effect on late endosomal morphology.

### ECHIDNA is involved in the local accumulation of root cap mucilage

**Fig. 4.**
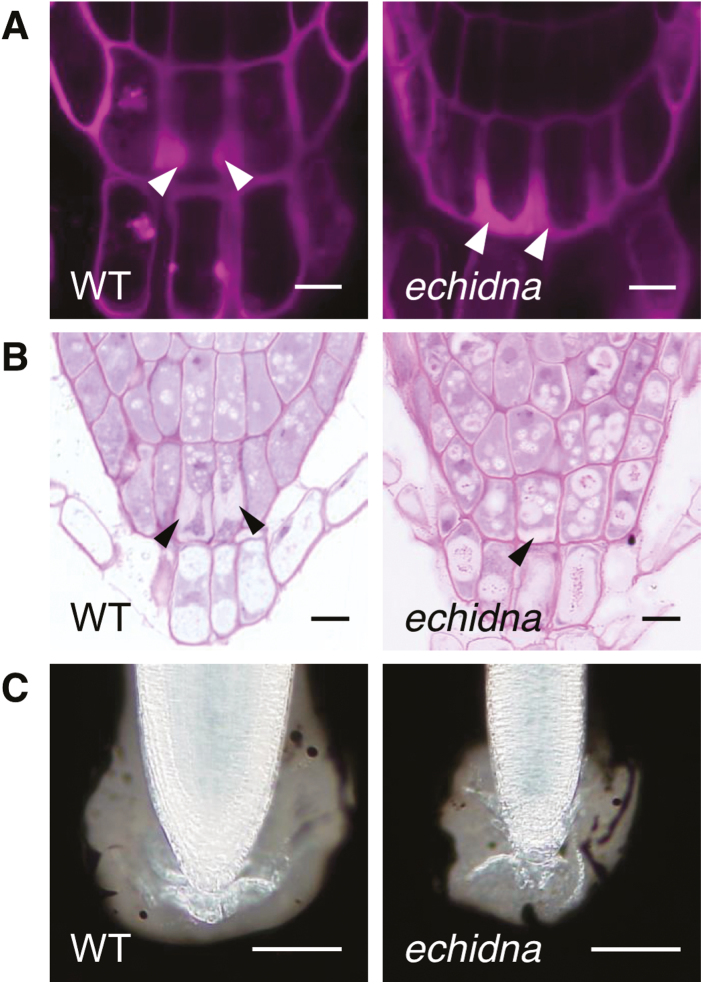
Root cap mucilage in the *echidna* mutant. (A) Confocal microscopic images of the root tip of the wild-type (WT) and *echidna* mutant stained using propidium iodide. (B) Toluidine blue-stained sections of the root tip of the WT and *echidna* mutant. (C) Images of the root tip of the WT and *echidna* mutant stained using india ink. Arrowheads indicate periplasmic mucilage. Bars=10 µm (A, B) and 50 µm (C).

Seed coat mucilage, a kind of polysaccharide that is secreted to the apoplast, has been shown to be inappropriately located in the vacuole in *echidna* seed coat cells (Gendre *et al.*, 2013; McFarlane *et al.*, 2013). We recently established efficient methods to detect root cap mucilage in Arabidopsis (Maeda *et al.*, 2019), and so we investigated the formation and secretion of root cap mucilage in *echidna* roots. First, we detected the periplasmic mucilage in wild-type and *echidna* seedling roots by staining with the fluorescent agent propidium iodide. Whereas periplasmic mucilage accumulated solely at the lateral side of columella cells in wild-type roots, it accumulated on the lower side and lateral side of columella cells in *echidna* roots (Fig. 4A). This defect in the distribution of periplasmic mucilage in *echidna* columella cells was confirmed by staining cross sections of root tips with toluidine blue (Fig. 4B). Next, we quantified the amount of root cap mucilage by staining with india ink. The area devoid of india ink in *echidna* seedling roots was significantly smaller than that in wild type roots (Fig. 4C and Supplementary Fig. S5), suggesting that *echidna* roots secrete less mucilage than wild-type roots. This may be due to the smaller size of *echidna* roots rather than a secretion defect in *echidna* cells. Our results suggest that ECHIDNA is required for both the proper transport and local accumulation of root cap mucilage in the lateral side of columella cells.

### *ECHIDNA* has a genetic interaction with *GFS9*

As described above, the *echidna* mutant has an effect on seed coloration, vacuolar trafficking (Fig. 1), and vacuolar morphology (Fig. 2). Similar phenotypes have been observed in mutant cells lacking GFS9, which is a Golgi-localized protein responsible for several membrane trafficking events in the plant endomembrane system, such as protein trafficking to vacuoles, vacuolar development, and endosomal maturation (Ichino *et al.*, 2014). In *gfs9* seeds, the contents of proanthocyanidins and flavonols are reduced, resulting in tan-colored seeds (Ichino *et al.*, 2014). Vacuolar proteins are missorted to outside cells in *gfs9* seeds, similar to *echidna*: the vacuolar marker SP-GFP-CT24 and the endogenous vacuolar protein 12S globulin were secreted to the apoplast in dry seeds of the *gfs9* mutant (Ichino *et al.*, 2014). In addition, a number of smaller vacuoles are present in *gfs9* cells (Ichino *et al.*, 2014). These findings prompted us to investigate the relationship between ECHIDNA and GFS9.

**Fig. 5.**
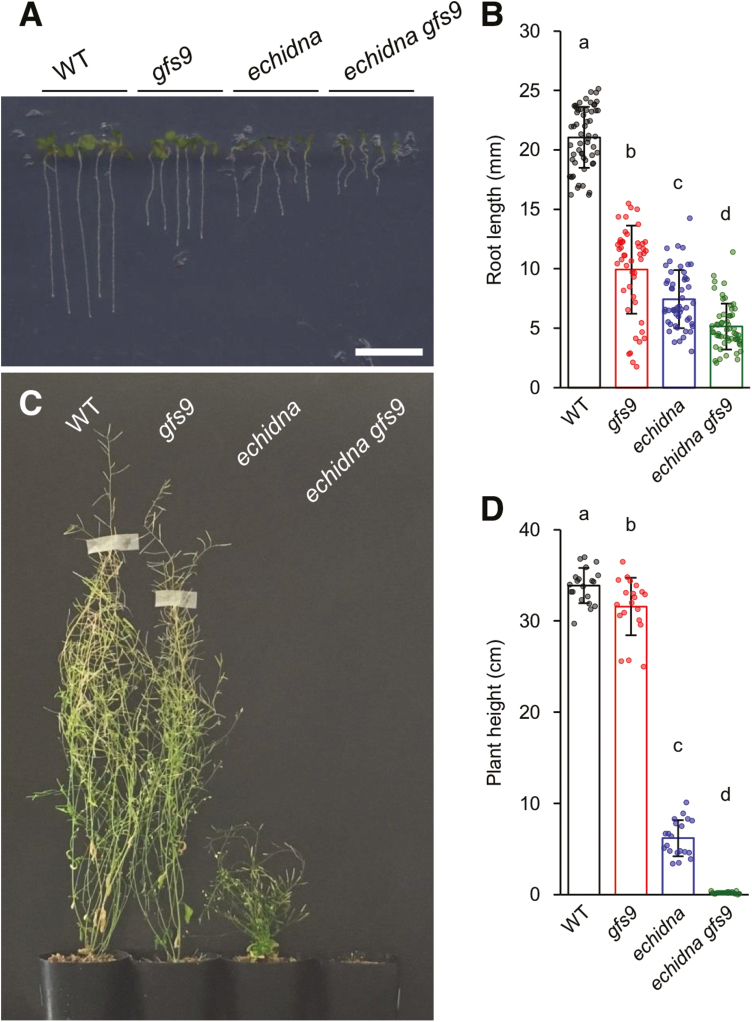
Plant growth of *echidna*, *gfs9*, and the *echidna gfs9* double mutant. (A) Image of 5-day-old seedlings. Bar=1 cm. (B) Primary root length of 5-day-old seedlings. The numbers of measured seedlings of wild-type (WT), *gfs9*, *echidna*, and *echidna gfs9* were 56, 47, 51, and 56, respectively. (C) Image of 80-day-old fully mature adult plants. (D) Plant height of 51-day-old adult plants; 20 individuals of each genotype were measured. Data represent the mean ±SD and raw data points. Different letters above the bars indicate statistically significant differences according to Tukey’s HSD test for multiple comparisons (α=0.01).

We crossed *echidna* and *gfs9* to generate double mutant plants. The *echidna* mutant showed severe defects in plant growth, with a shorter seedling root, and a bushy and dwarf plant architecture (Fig. 5) (Gendre *et al.*, 2011; Fan *et al.*, 2014). The *gfs9* mutant also had a shorter seedling root, a slightly shorter plant height, and a smaller plant size than the wild type, but this phenotype was not as severe as that of the *echidna* mutant (Fig. 5 and Supplementary Fig. S6). The seedlings of the established *echidna gfs9* double mutant had a much shorter root length than that of either of the single mutants (Fig. 5A, B). At the mature adult stage, the *echidna gfs9* double mutant plant had severe growth defects and it did not produce fully expanded rosette leaves (Supplementary Fig. S6A, B). The plant height and size of the *echidna gfs9* double mutant were smaller than those of either of the single mutants (Fig. 5C, D and Supplementary Fig. S6A, B). Additionally, the double mutant was frequently unsuccessful in bolting inflorescence and flowering (Supplementary Fig. S6C, D), which resulted in defective seed production. These results demonstrated a synthetic genetic interaction between *ECHIDNA* and *GFS9* in plant growth.

## Discussion

In this study, we describe new roles of Arabidopsis ECHIDNA protein in seed coloration, protein trafficking to the vacuole (Fig. 1), and vacuolar development (Fig. 2). The absence of staining with *p*-dimethylaminocinnamaldehyde and tan color of *echidna* seeds suggest lower accumulation of proanthocyanidins, a type of flavonoid (Fig. 1A, B). In the flavonoid biosynthetic pathway, early biosynthetic steps are performed on the ER membrane, where multi-enzyme complexes for flavonoid biosynthesis, including chalcone synthase and chalcone isomerase, are located (Saslowsky and Winkel-Shirley, 2001; Jørgensen *et al.*, 2005). Finally, most of the synthesized flavonoids accumulate in the vacuole. It is not yet known how the membrane trafficking component ECHIDNA affects the transport and accumulation of flavonoids. One possibility may be that vacuolar biogenesis, mediated by ECHIDNA, contributes to flavonoid accumulation in the vacuole. In the *echidna* mutant, invagination of mCherry-VAMP711-labels on the vacuolar membrane and aggregation with multiple membranes was evident in both hypocotyl and root cells (Fig. 2). A close connection between vacuolar morphology and flavonoid accumulation has been reported in Arabidopsis. Multiple smaller vacuoles have been found in the seed coat cells, where proanthocyanidins are accumulated, of several flavonoid-deficient mutants, including a MATE-type transporter mutant *tt12* (Debeaujon *et al.*, 2001), P_3A_-type H^+^-ATPase mutant *tt13*/*aha10* (Baxter *et al.*, 2005; Appelhagen *et al.*, 2015), leucoanthocyanidin dioxygenase mutant *tt18*/*tannin deficient seed 4* (Abrahams *et al.*, 2003), and glutathione-*S*-transferase mutant *tt19* (Kitamura *et al.*, 2004). In contrast, the chalcone synthase mutant *tt4* has a defect in vacuolar biogenesis in hypocotyl cells (Rosado *et al.*, 2011), which implies that the flavonoid itself has effects on vacuolar development and integrity.

**Fig. 6.**
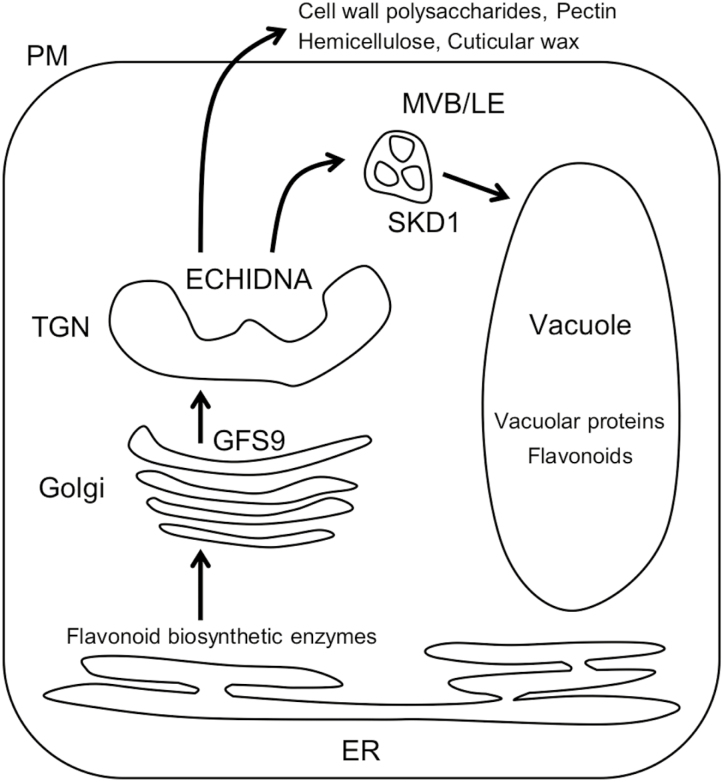
Model of the intracellular trafficking pathway mediated by ECHIDNA. Early steps of flavonoid biosynthesis are performed on the endoplasmic reticulum (ER), and the final products (e.g. proanthocyanidins) accumulate in the vacuole. Vacuolar proteins (e.g. seed storage proteins) are also transported from the synthetic site on the ER to the final destination vacuole. GREEN FLUORESCENT SEED 9 (GFS9) on the Golgi apparatus and ECHIDNA on the *trans*-Golgi network (TGN) play roles in the trafficking of vacuolar proteins and flavonoids to the vacuoles. ECHIDNA also mediates the secretion of cell wall components and cuticular wax. SUPPRESSOR OF K^+^ TRANSPORT GROWTH DEFFECT1 (SKD1), a component of the ESCRT machinery on the multivesicular body (MVB)/late endosome (LE), has also been reported to be involved in the vacuolar trafficking of proteins and seed coat pigmentation. PM, plasma membrane.

Another possibility is that ECHIDNA-mediated trafficking machinery is involved in flavonoid transport to the vacuole. The lack of *ECHIDNA* might cause missorting of flavonoids and, therefore, lead to reduced accumulation levels in the vacuole. It is possible that vacuolar proteins and flavonoids share the trafficking pathway from the ER to vacuole via the TGN, where the ECHIDNA protein is located. This idea is consistent with previous reports showing that the protein trafficking route from ER to vacuole contributes to the vacuolar accumulation of anthocyanin (Poustka *et al.*, 2007; Rosado *et al.*, 2011). To date, two membrane trafficking components, GFS9 and SUPPRESSOR OF K^+^ TRANSPORT GROWTH DEFFECT1 (SKD1), have been reported to affect seed coat pigmentation (Fig. 6). The Arabidopsis mutant lacking GFS9, which is associated with the Golgi apparatus, showed multiple phenotypes, including disrupted vacuolar protein sorting and the *transparent testa* phenotype (Ichino *et al.*, 2014). In addition, Arabidopsis plants expressing a dominant-negative version of SKD1, which is a subunit of the ESCRT machinery localized in the multivesicular body, showed defects in vacuolar protein sorting and seed coat pigmentation (Shahriari *et al.*, 2010a, b). These observations suggest that seed coat pigmentation might be dependent on membrane trafficking to the vacuole.

In addition to the above hypotheses, we cannot rule out the possibility that the seed color phenotype in the *echidna* mutant is caused by a defect in flavonoid biosynthesis on the ER. Previously, reduced wax biosynthesis has been reported in the defective mutant of ROOT HAIR DEFECTIVE3, which is responsible for ER organization (McFarlane *et al.*, 2014). Wax biosynthesis occurs in the ER, similar to flavonoid biosynthesis (Lee and Suh, 2013). ECHIDNA also affects ER morphology (McFarlane *et al.*, 2014). It is possible that the defective ER morphology in the *echidna* mutant disturbs flavonoid biosynthesis due to the disorganization of flavonoid metabolic enzymes on defective ER membranes.

In this study, we found a genetic interaction between *ECHIDNA* and *GFS9* (Fig. 5). The single mutants lacking either gene displayed multiple defects including plant growth, seed coloration, vacuolar trafficking, and vacuolar morphology. The *echidna gfs9* double mutant showed more severe defects in plant growth than either the *echidna* or the *gfs9* mutant alone (Fig. 5). This synthetic genetic interaction between *ECHIDNA* and *GFS9* in plant growth suggests two possibilities: either the two genes act in parallel pathways that converge at a node, or they act in successive steps of the same pathway (Pérez-Pérez *et al.*, 2009). ECHIDNA and GFS9 might act in two different pathways for trafficking to the vacuole. Alternatively, ECHIDNA and GFS9 might function in the same vacuolar trafficking pathway, residing at distinct compartments: ECHIDNA at the TGN and GFS9 at the Golgi apparatus (Fig. 6). Probably due to their different locations, ECHIDNA and GFS9 have different effects on vacuolar morphology. The *echidna* mutant has a single central vacuole with defective membrane dynamics (Fig. 2), while the *gfs9* mutant has multiple smaller vacuoles (Ichino *et al.*, 2014). Moreover, the aggregation in *echidna* showed vacuolar membrane identity (Fig. 2), while the aggregation in *gfs9* consists of enlarged late endosomes (Ichino *et al.*, 2014). The different effects of ECHIDNA and GFS9 on vacuolar development could be explained by their distinct functions. ECHIDNA has been shown to maintain TGN integrity (Gendre *et al.*, 2011; Boutte *et al*., 2013). The lack of ECHIDNA caused the mislocalization of several TGN-localized proteins, including vacuolar H^+^-ATPase subunit a1 (Gendre *et al.*, 2011). TGN integrity may underlie sequential vacuolar development, because plant endomembrane compartments originate and connect with each other. In plants, the TGN is derived from and associated with the Golgi apparatus (Staehelin and Kang, 2008; Uemura *et al.*, 2014), and a subdomain of the TGN matures to become a multivesicular body or late endosome (Scheuring *et al.*, 2011; Singh *et al.*, 2014). In contrast, the *gfs9* mutant may be defective in membrane fusion to the vacuole, because the GFS9 orthologue of *Drosophila melanogaster* interacts with a subunit of the vacuolar tethering complex HOPS (Kim *et al.*, 2010). The HOPS complex is required for membrane fusion on the vacuole in Arabidopsis (Brillada *et al.*, 2018; Takemoto *et al.*, 2018).

We report a novel finding of the involvement of ECHIDNA in vacuolar trafficking of proteins (Fig. 1C–E). Together with the well-established function of ECHIDNA in protein secretion (Gendre *et al.*, 2011; Boutte *et al*., 2013), ECHIDNA has roles in distinct pathways—the vacuolar trafficking pathway and the secretory pathway— in the intracellular trafficking network of plant cells (Fig. 6). Misdistribution of SP-GFP-CT24 to the extracellular space was found in *echidna* seed cells (Fig. 1D), even though ECHIDNA is involved in secretion. This finding implies that the *echidna* mutant still has some activity in secretion. A previous report suggested that ECHIDNA is required for the route of secretory traffic defined by the secretion reporter secGFP but not for all secretory cargos (Gendre *et al.*, 2011). Consistent with this notion, we found secretion of root cap mucilage from *echidna* mutant cells (Fig. 4). Moreover, the *echidna* mutant cell is able to form a cell wall, the construction of which is supported by the secretion of cell wall components.

The roles of ECHIDNA in the vacuolar and secretory trafficking pathways account for phytochemicals as well as proteins. Similar to its role in the vacuolar accumulation of flavonoids, ECHIDNA is involved in the secretion of cell wall polysaccharides (Fig. 4), pectin, hemicellulose (Gendre *et al.*, 2013; McFarlane *et al.*, 2013), and cuticular wax (McFarlane *et al.*, 2014). The molecular mechanism underlying the secretion of cell wall components has not been completely determined. Considering that ECHIDNA is required for multidirectional protein trafficking pathways, a similar mechanism may operate in ECHIDNA-mediated flavonoid accumulation in the vacuole and ECHIDNA-mediated secretion of cell wall components to the exterior of the cell. In fact, misdistribution of root cap mucilage (Fig. 4) and of pectin within the vacuole and ER (McFarlane *et al.*, 2013) has been observed in *echidna* seedling root and seed coat cells, respectively. This raises the possibility of misdistribution of flavonoids in *echidna* seed coat cells.

Our findings reveal that ECHIDNA has a role in multidirectional pathways of the intracellular trafficking network: the vacuolar trafficking pathway and the secretory pathway (Fig. 6). We report a new role of the plant TGN in vacuolar development and in the distribution of specialized metabolites of plants, including cell wall components and seed flavonoids. In addition, we show that ECHIDNA-mediated trafficking at the TGN and GFS9-mediated trafficking at the Golgi apparatus in tandem contribute to vacuolar trafficking and vacuolar development, which are required for flavonoid accumulation in plant seeds and for plant growth. Further research concerning the intracellular dynamics of plant specialized metabolites could result in the discovery of new roles of intracellular membrane compartments and networks.

## Supplementary data

Supplementary data are available at *JXB* online.

Fig. S1. Confocal images of SP-GFP-CT24 in *echidna* seedlings.

Fig. S2. Confocal images of aberrant structures labeled with mCherry-VAMP711 in *echidna* hypocotyl cells.

Fig. S3. Quantitative analysis of protein storage vacuoles in *echidna* dry seeds.

Fig. S4. Confocal images and quantitative analysis of mCherry-ARA7-labeled late endosomes.

Fig. S5. Representative images converted by Trainable Weka segmentation and quantitative analysis of india ink staining.

Fig. S6. Plant size and reproductive growth of the *echidna gfs9* double mutant.

eraa147_suppl_Supplementary_Figures_S1-S6

## Abbreviations

ER: endoplasmic reticulum
GFP: green fluorescent protein
GFS: GREEN FLUORESCENT SEED
PSV: protein storage vacuole
TGN: *trans*-Golgi network.

## References

[CIT0001] Abrahams S, Lee E, Walker AR, Tanner GJ, Larkin PJ, Ashton AR 2003 The *Arabidopsis TDS4* gene encodes leucoanthocyanidin dioxygenase (LDOX) and is essential for proanthocyanidin synthesis and vacuole development. The Plant Journal 35, 624–636. 12940955 10.1046/j.1365-313x.2003.01834.x

[CIT0002] Abrahams S, Tanner GJ, Larkin PJ, Ashton AR 2002 Identification and biochemical characterization of mutants in the proanthocyanidin pathway in Arabidopsis. Plant Physiology 130, 561–576. 12376625 10.1104/pp.006189 PMC166587

[CIT0003] Appelhagen I, Nordholt N, Seidel T, Spelt K, Koes R, Quattrochio F, Sagasser M, Weisshaar B 2015 TRANSPARENT TESTA 13 is a tonoplast P_3A_-ATPase required for vacuolar deposition of proanthocyanidins in *Arabidopsis thaliana* seeds. The Plant Journal 82, 840–849. 25891958 10.1111/tpj.12854

[CIT0004] Arganda-Carreras I, Kaynig V, Rueden C, Eliceiri KW, Schindelin J, Cardona A, Sebastian Seung H 2017 Trainable Weka Segmentation: a machine learning tool for microscopy pixel classification. Bioinformatics 33, 2424–2426. 28369169 10.1093/bioinformatics/btx180

[CIT0005] Baxter IR, Young JC, Armstrong G, Foster N, Bogenschutz N, Cordova T, Peer WA, Hazen SP, Murphy AS, Harper JF 2005 A plasma membrane H^+^-ATPase is required for the formation of proanthocyanidins in the seed coat endothelium of *Arabidopsis thaliana*. Proceedings of the National Academy of Sciences, USA 102, 2649–2654. 10.1073/pnas.0406377102 PMC548969 15695592

[CIT0006] Beachy RN, Chen ZL, Horsch RB, Rogers SG, Hoffmann NJ, Fraley RT 1985 Accumulation and assembly of soybean β-conglycinin in seeds of transformed petunia plants. The EMBO Journal 4, 3047–3053. 16453646 10.1002/j.1460-2075.1985.tb04044.x PMC554621

[CIT0007] Boutté Y, Jonsson K, McFarlane HE, Johnson E, Gendre D, Swarup R, Friml J, Samuels L, Robert S, Bhalerao RP 2013 ECHIDNA-mediated post-Golgi trafficking of auxin carriers for differential cell elongation. Proceedings of the National Academy of Sciences, USA 110, 16259–16264. 10.1073/pnas.1309057110 PMC3791722 24043780

[CIT0008] Brillada C, Zheng J, Krüger F, Rovira-Diaz E, Askani JC, Schumacher K, Rojas-Pierce M 2018 Phosphoinositides control the localization of HOPS subunit VPS41, which together with VPS33 mediates vacuole fusion in plants. Proceedings of the National Academy of Sciences, USA 115, E8305–E8314. 10.1073/pnas.1807763115 PMC6126739 30104351

[CIT0009] Chanoca A, Kovinich N, Burkel B, Stecha S, Bohorquez-Restrepo A, Ueda T, Eliceiri KW, Grotewold E, Otegui MS 2015 Anthocyanin vacuolar inclusions form by a microautophagy mechanism. The Plant Cell 27, 2545–2559. 26342015 10.1105/tpc.15.00589 PMC4815043

[CIT0010] Debeaujon I, Peeters AJ, Léon-Kloosterziel KM, Koornneef M 2001 The *TRANSPARENT TESTA12* gene of Arabidopsis encodes a multidrug secondary transporter-like protein required for flavonoid sequestration in vacuoles of the seed coat endothelium. The Plant Cell 13, 853–871. 11283341 10.1105/tpc.13.4.853 PMC135529

[CIT0011] Drakakaki G, van de Ven W, Pan S, et al 2012 Isolation and proteomic analysis of the SYP61 compartment reveal its role in exocytic trafficking in *Arabidopsis*. Cell Research 22, 413–424. 21826108 10.1038/cr.2011.129 PMC3271593

[CIT0012] Fan X, Yang C, Klisch D, Ferguson A, Bhaellero RP, Niu X, Wilson ZA 2014 ECHIDNA protein impacts on male fertility in *Arabidopsis* by mediating trans-Golgi network secretory trafficking during anther and pollen development. Plant Physiology 164, 1338–1349. 24424320 10.1104/pp.113.227769 PMC3938624

[CIT0013] Francisco RM, Regalado A, Ageorges A, et al 2013 ABCC1, an ATP binding cassette protein from grape berry, transports anthocyanidin 3-*O*-glucosides. The Plant Cell 25, 1840–1854. 23723325 10.1105/tpc.112.102152 PMC3694709

[CIT0014] Fuji K, Shimada T, Takahashi H, Tamura K, Koumoto Y, Utsumi S, Nishizawa K, Maruyama N, Hara-Nishimura I 2007 *Arabidopsis* vacuolar sorting mutants (*green fluorescent seed*) can be identified efficiently by secretion of vacuole-targeted green fluorescent protein in their seeds. The Plant Cell 19, 597–609. 17293568 10.1105/tpc.106.045997 PMC1867321

[CIT0015] Geldner N, Dénervaud-Tendon V, Hyman DL, Mayer U, Stierhof YD, Chory J 2009 Rapid, combinatorial analysis of membrane compartments in intact plants with a multicolor marker set. The Plant Journal 59, 169–178. 19309456 10.1111/j.1365-313X.2009.03851.x PMC4854200

[CIT0016] Gendre D, McFarlane HE, Johnson E, Mouille G, Sjödin A, Oh J, Levesque-Tremblay G, Watanabe Y, Samuels L, Bhalerao RP 2013 *Trans*-Golgi network localized ECHIDNA/Ypt interacting protein complex is required for the secretion of cell wall polysaccharides in *Arabidopsis*. The Plant Cell 25, 2633–2646. 23832588 10.1105/tpc.113.112482 PMC3753388

[CIT0017] Gendre D, Oh J, Boutté Y, et al 2011 Conserved *Arabidopsis* ECHIDNA protein mediates *trans*-Golgi-network trafficking and cell elongation. Proceedings of the National Academy of Sciences, USA 108, 8048–8053. 10.1073/pnas.1018371108 PMC3093476 21512130

[CIT0018] Gomez C, Terrier N, Torregrosa L, et al 2009 Grapevine MATE-type proteins act as vacuolar H^+^-dependent acylated anthocyanin transporters. Plant Physiology 150, 402–415. 19297587 10.1104/pp.109.135624 PMC2675721

[CIT0019] Goodman CD, Casati P, Walbot V 2004 A multidrug resistance-associated protein involved in anthocyanin transport in *Zea mays*. The Plant Cell 16, 1812–1826. 15208386 10.1105/tpc.022574 PMC514163

[CIT0020] Grotewold E 2006 The genetics and biochemistry of floral pigments. Annual Review of Plant Biology 57, 761–780. 10.1146/annurev.arplant.57.032905.105248 16669781

[CIT0021] Hawes MC, Gunawardena U, Miyasaka S, Zhao X 2000 The role of root border cells in plant defense. Trends in Plant Science 5, 128–133. 10707079 10.1016/s1360-1385(00)01556-9

[CIT0022] Hsieh K, Huang AH 2007 Tapetosomes in *Brassica* tapetum accumulate endoplasmic reticulum-derived flavonoids and alkanes for delivery to the pollen surface. The Plant Cell 19, 582–596. 17307923 10.1105/tpc.106.049049 PMC1867322

[CIT0023] Ichino T, Fuji K, Ueda H, et al 2014 GFS9/TT9 contributes to intracellular membrane trafficking and flavonoid accumulation in *Arabidopsis thaliana*. The Plant Journal 80, 410–423. 25116949 10.1111/tpj.12637

[CIT0024] Jørgensen K, Rasmussen AV, Morant M, Nielsen AH, Bjarnholt N, Zagrobelny M, Bak S, Møller BL 2005 Metabolon formation and metabolic channeling in the biosynthesis of plant natural products. Current Opinion in Plant Biology 8, 280–291. 15860425 10.1016/j.pbi.2005.03.014

[CIT0025] Kevan PG, Baker HG 1983 Insects as flower visitors and pollinators. Annual Review of Entomology 28, 407–453.

[CIT0026] Kim S, Wairkar YP, Daniels RW, DiAntonio A 2010 The novel endosomal membrane protein Ema interacts with the class C Vps–HOPS complex to promote endosomal maturation. Journal of Cell Biology 188, 717–734. 20194640 10.1083/jcb.200911126 PMC2835942

[CIT0027] Kitamura S, Shikazono N, Tanaka A 2004 *TRANSPARENT TESTA 19* is involved in the accumulation of both anthocyanins and proanthocyanidins in *Arabidopsis*. The Plant Journal 37, 104–114. 14675436 10.1046/j.1365-313x.2003.01943.x

[CIT0028] Kulich I, Pečenková T, Sekereš J, Smetana O, Fendrych M, Foissner I, Höftberger M, Zárský V 2013 Arabidopsis exocyst subcomplex containing subunit EXO70B1 is involved in autophagy-related transport to the vacuole. Traffic 14, 1155–1165. 23944713 10.1111/tra.12101

[CIT0029] Lee SB, Suh MC 2013 Recent advances in cuticular wax biosynthesis and its regulation in *Arabidopsis*. Molecular Plant 6, 246–249. 23253604 10.1093/mp/sss159

[CIT0030] Lepiniec L, Debeaujon I, Routaboul JM, Baudry A, Pourcel L, Nesi N, Caboche M 2006 Genetics and biochemistry of seed flavonoids. Annual Review of Plant Biology 57, 405–430. 10.1146/annurev.arplant.57.032905.105252 16669768

[CIT0031] Maeda K, Kunieda T, Tamura K, Hatano K, Hara-Nishimura I, Shimada T 2019 Identification of periplasmic root-cap mucilage in developing columella cells of *Arabidopsis thaliana*. Plant & Cell Physiology 60, 1296–1303. 30892660 10.1093/pcp/pcz047

[CIT0032] Marinova K, Pourcel L, Weder B, Schwarz M, Barron D, Routaboul JM, Debeaujon I, Klein M 2007 The *Arabidopsis* MATE transporter TT12 acts as a vacuolar flavonoid/H^+^-antiporter active in proanthocyanidin-accumulating cells of the seed coat. The Plant Cell 19, 2023–2038. 17601828 10.1105/tpc.106.046029 PMC1955721

[CIT0033] McFarlane HE, Watanabe Y, Gendre D, Carruthers K, Levesque-Tremblay G, Haughn GW, Bhalerao RP, Samuels L 2013 Cell wall polysaccharides are mislocalized to the vacuole in *echidna* mutants. Plant & Cell Physiology 54, 1867–1880. 24058145 10.1093/pcp/pct129

[CIT0034] McFarlane HE, Watanabe Y, Yang W, Huang Y, Ohlrogge J, Samuels AL 2014 Golgi- and trans-Golgi network-mediated vesicle trafficking is required for wax secretion from epidermal cells. Plant Physiology 164, 1250–1260. 24468625 10.1104/pp.113.234583 PMC3938617

[CIT0035] Nishimura M, Takeuchi Y, Debellis L, Hara-nishimura I 1993 Leaf peroxisomes are directly transformed to glyoxysomes during senescence of pumpkin cotyledons. Protoplasma 175, 131–137.

[CIT0036] Nishizawa K, Maruyama N, Satoh R, Fuchikami Y, Higasa T, Utsumi S 2003 A C-terminal sequence of soybean β-conglycinin α′ subunit acts as a vacuolar sorting determinant in seed cells. The Plant Journal 34, 647–659. 12787246 10.1046/j.1365-313x.2003.01754.x

[CIT0037] Pérez-Pérez JM, Candela H, Micol JL 2009 Understanding synergy in genetic interactions. Trends in Genetics 25, 368–376. 19665253 10.1016/j.tig.2009.06.004

[CIT0038] Pourcel L, Irani NG, Lu Y, Riedl K, Schwartz S, Grotewold E 2010 The formation of anthocyanic vacuolar inclusions in *Arabidopsis thaliana* and implications for the sequestration of anthocyanin pigments. Molecular Plant 3, 78–90. 20085894 10.1093/mp/ssp071 PMC2807924

[CIT0039] Poustka F, Irani NG, Feller A, Lu Y, Pourcel L, Frame K, Grotewold E 2007 A trafficking pathway for anthocyanins overlaps with the endoplasmic reticulum-to-vacuole protein-sorting route in Arabidopsis and contributes to the formation of vacuolar inclusions. Plant Physiology 145, 1323–1335. 17921343 10.1104/pp.107.105064 PMC2151709

[CIT0040] R Core Team 2016 R: A language and environment for statistical computing. Vienna: R Foundation for Statistical Computing https://www.R-project.org/

[CIT0041] Ravikumar R, Kalbfuss N, Gendre D, et al 2018 Independent yet overlapping pathways ensure the robustness and responsiveness of trans-Golgi network functions in *Arabidopsis*. Development 145, dev169201. 30404777 10.1242/dev.169201

[CIT0042] Rosado A, Hicks GR, Norambuena L, et al 2011 Sortin1-hypersensitive mutants link vacuolar-trafficking defects and flavonoid metabolism in *Arabidopsis* vegetative tissues. Chemistry & Biology 18, 187–197. 21338917 10.1016/j.chembiol.2010.11.015

[CIT0043] Rounds CM, Lubeck E, Hepler PK, Winship LJ 2011 Propidium iodide competes with Ca^2+^ to label pectin in pollen tubes and Arabidopsis root hairs. Plant Physiology 157, 175–187. 21768649 10.1104/pp.111.182196 PMC3165868

[CIT0044] Routaboul JM, Kerhoas L, Debeaujon I, Pourcel L, Caboche M, Einhorn J, Lepiniec L 2006 Flavonoid diversity and biosynthesis in seed of *Arabidopsis thaliana*. Planta 224, 96–107. 16395586 10.1007/s00425-005-0197-5

[CIT0045] Saslowsky D, Winkel-Shirley B 2001 Localization of flavonoid enzymes in Arabidopsis roots. The Plant Journal 27, 37–48. 11489181 10.1046/j.1365-313x.2001.01073.x

[CIT0046] Scheuring D, Viotti C, Krüger F, et al 2011 Multivesicular bodies mature from the *trans*-Golgi network/early endosome in *Arabidopsis*. The Plant Cell 23, 3463–3481. 21934143 10.1105/tpc.111.086918 PMC3203422

[CIT0047] Shahriari M, Hülskamp M, Schellmann S 2010 Seeds of Arabidopsis plants expressing dominant-negative AtSKD1 under control of the *GL2* promoter show a *transparent testa* phenotype and a mucilage defect. Plant Signaling & Behavior 5, 1308–1310. 20930567 10.4161/psb.5.10.13134 PMC3115375

[CIT0048] Shahriari M, Keshavaiah C, Scheuring D, Sabovljevic A, Pimpl P, Häusler RE, Hülskamp M, Schellmann S 2010 The AAA-type ATPase AtSKD1 contributes to vacuolar maintenance of *Arabidopsis thaliana*. The Plant Journal 64, 71–85. 20663085 10.1111/j.1365-313X.2010.04310.x

[CIT0049] Sheehan H, Moser M, Klahre U, Esfeld K, Dell’Olivo A, Mandel T, Metzger S, Vandenbussche M, Freitas L, Kuhlemeier C 2016 *MYB-FL* controls gain and loss of floral UV absorbance, a key trait affecting pollinator preference and reproductive isolation. Nature Genetics 48, 159–166. 26656847 10.1038/ng.3462

[CIT0050] Shimada T, Fuji K, Tamura K, Kondo M, Nishimura M, Hara-Nishimura I 2003 Vacuolar sorting receptor for seed storage proteins in *Arabidopsis thaliana*. Proceedings of the National Academy of Sciences, USA 100, 16095–16100. 10.1073/pnas.2530568100 PMC307698 14657332

[CIT0051] Shimada T, Yamada K, Kataoka M, et al 2003 Vacuolar processing enzymes are essential for proper processing of seed storage proteins in *Arabidopsis thaliana*. Journal of Biological Chemistry 278, 32292–32299. 12799370 10.1074/jbc.M305740200

[CIT0052] Shirley BW, Kubasek WL, Storz G, Bruggemann E, Koornneef M, Ausubel FM, Goodman HM 1995 Analysis of *Arabidopsis* mutants deficient in flavonoid biosynthesis. The Plant Journal 8, 659–671. 8528278 10.1046/j.1365-313x.1995.08050659.x

[CIT0053] Singh MK, Krüger F, Beckmann H, et al 2014 Protein delivery to vacuole requires SAND protein-dependent Rab GTPase conversion for MVB-vacuole fusion. Current Biology 24, 1383–1389. 24881875 10.1016/j.cub.2014.05.005

[CIT0054] Staehelin LA, Kang BH 2008 Nanoscale architecture of endoplasmic reticulum export sites and of Golgi membranes as determined by electron tomography. Plant Physiology 147, 1454–1468. 18678738 10.1104/pp.108.120618 PMC2492626

[CIT0055] Sugiyama A, Shitan N, Yazaki K 2007 Involvement of a soybean ATP-binding cassette-type transporter in the secretion of genistein, a signal flavonoid in legume-*Rhizobium* symbiosis. Plant Physiology 144, 2000–2008. 17556512 10.1104/pp.107.096727 PMC1949875

[CIT0056] Takemoto K, Ebine K, Askani JC, Krüger F, Gonzalez ZA, Ito E, Goh T, Schumacher K, Nakano A, Ueda T 2018 Distinct sets of tethering complexes, SNARE complexes, and Rab GTPases mediate membrane fusion at the vacuole in Arabidopsis. Proceedings of the National Academy of Sciences, USA 115, E2457–E2466. 10.1073/pnas.1717839115 PMC5877921 29463724

[CIT0057] Uemura T, Suda Y, Ueda T, Nakano A 2014 Dynamic behavior of the *trans*-golgi network in root tissues of Arabidopsis revealed by super-resolution live imaging. Plant & Cell Physiology 55, 694–703. 24443496 10.1093/pcp/pcu010

[CIT0058] Zhao J, Dixon RA 2009 MATE transporters facilitate vacuolar uptake of epicatechin 3′-*O*-glucoside for proanthocyanidin biosynthesis in *Medicago truncatula* and *Arabidopsis*. The Plant Cell 21, 2323–2340. 19684242 10.1105/tpc.109.067819 PMC2751950

