# Supplementary material for: Arabidopsis ECHIDNA protein is involved in seed coloration, protein trafficking to vacuoles, and vacuolar biogenesis
Source: J Exp Bot. 2020 Mar 23;71(14):3999–4009. doi: 10.1093/jxb/eraa147 (PMC7475254; doi:10.1093/jxb/eraa147)
Supplement: eraa147_suppl_Supplementary_Figures_S1-S6 [file eraa147_suppl_supplementary_figures_s1-s6.pdf]

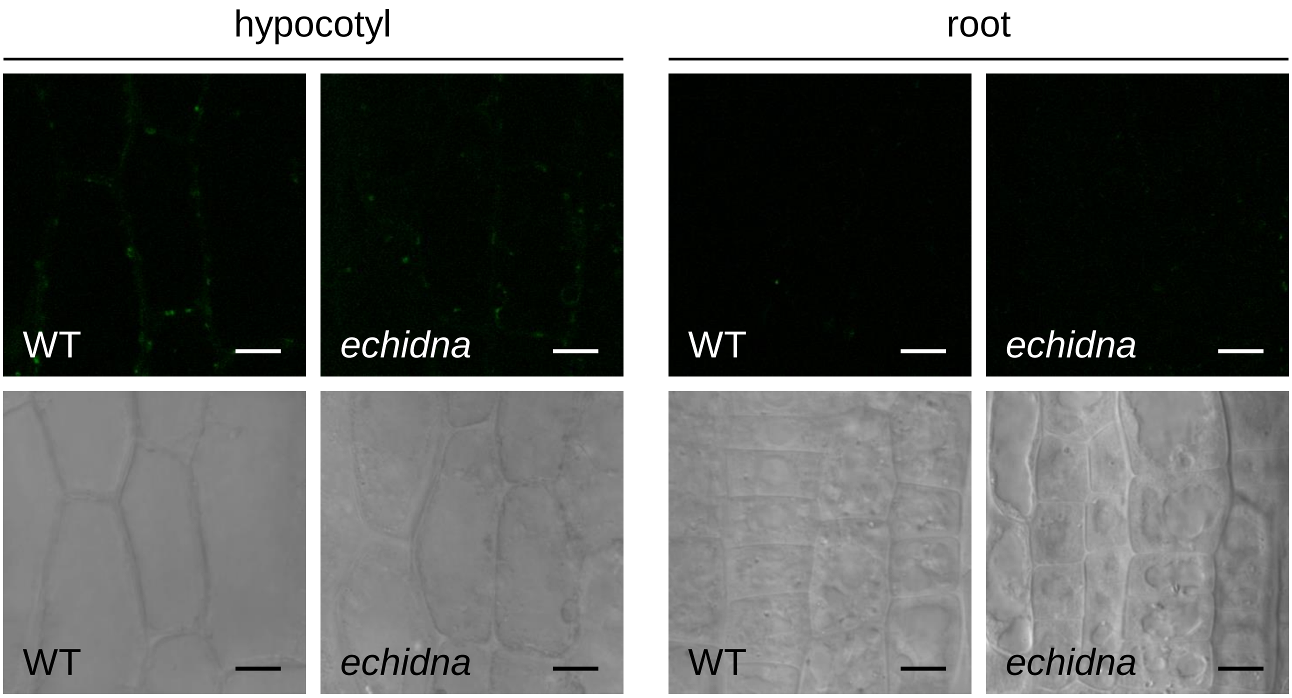

Fig. S1 Confocal images of SP-GFP-CT24 in the *echidna* seedlings. The fluorescence images of SP-GFP-CT24 (top) and corresponding DIC images (bottom) of seedling hypocotyl and roots in the wild type (WT) and *echidna* are shown. Bars, 10 μm.

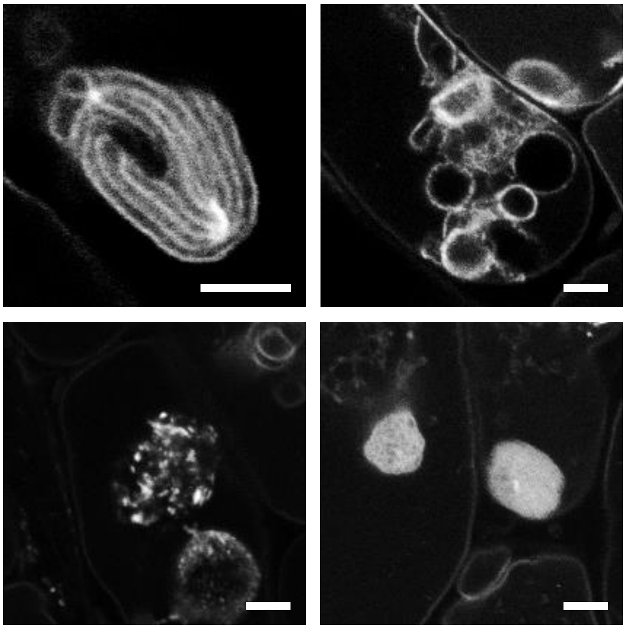

Fig. S2 Confocal images of aberrant structures labeled with mCherry-VAMP711 in *echidna* hypocotyl cells. Bars, 5 μm.

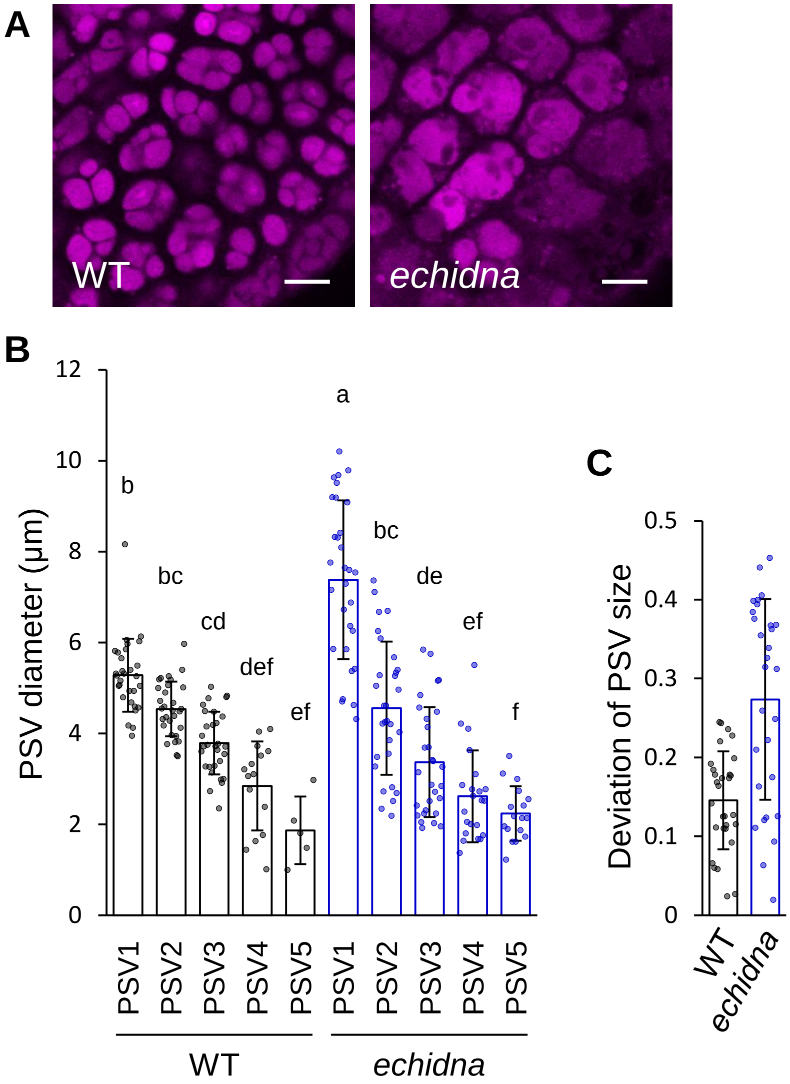

Fig. S3 Quantitative analysis of protein storage vacuoles (PSV) in *echidna* dry seeds. (A) Representative image for the quantitative analysis of PSV size. The auto-fluorescence of PSVs indicates PSV morphology in embryonic cotyledon cells of wild-type (WT) and *echidna* dry seeds. Bars, 10 µm. (B) PSV diameter in dry seeds. PSV1 is the largest PSV within a single cell, and PSV2 to PSV5 are the second to fifth largest PSVs within the same cell. Thirty embryonic cotyledon cells were measured in both WT and *echidna* dry seeds. Each plot represents the diameter of 30 PSV1, 30 PSV2, 30 PSV3, 15 PSV4, and 5 PSV5 in WT cells and 30 PSV1, 30 PSV2, 29 PSV3, 22 PSV4, and 17 PSV5 in *echidna* cells. (C) Deviation of PSV size. Standard deviation among the relative diameters of PSV1, PSV2, and PSV3 within a single cell is shown (relative PSV1 size in each cell was set as 1.0). Thirty and 29 embryonic cotyledon cells were measured in WT and *echidna* dry seeds, respectively. Data represent the average with S.D., and the raw data points are shown. Letters indicate statistical significance corresponding to Tukey's HSD test for multiple comparisons ($\alpha = 0.05$) (B), and the *p*-value ($p = 1.71E^{-5}$) was statistically calculated using Student's t-test (C).

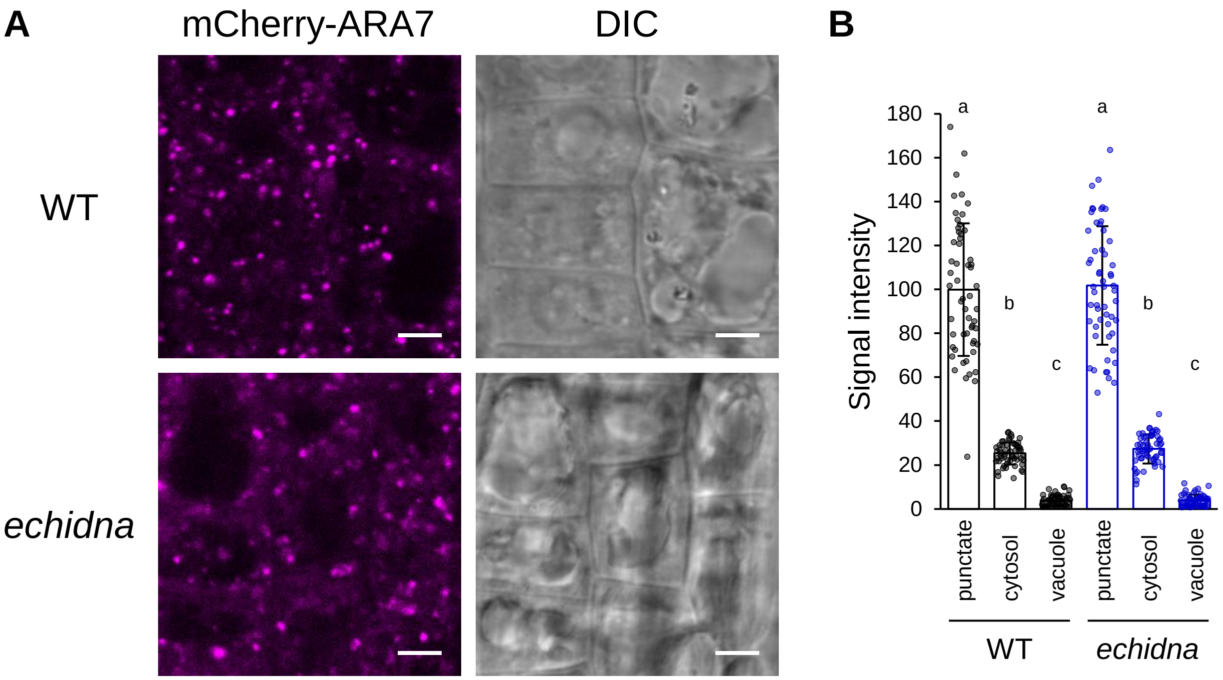

Fig. S4 Confocal images and quantitative analysis of mCherry-ARA7 labeled late endosomes. (A) Confocal images of mCherry-ARA7-labeled late endosomes in wild-type (WT) and *echidna* seedling roots. Corresponding DIC images of Fig. 3E and F. Bars, 5 μm. (B) Fluorescent signal intensities of punctate structures, cytosols, and vacuoles. Each plot represents the mean of four regions of interest within a single root cell of the elongation zone. Four cells per plant, 14 plants, and a total of 56 cells were measured for each genotype. Data represent the average of the 56 means with S.D. Letters indicate statistical significance corresponding to Tukey's HSD test for multiple comparisons (α = 0.05).

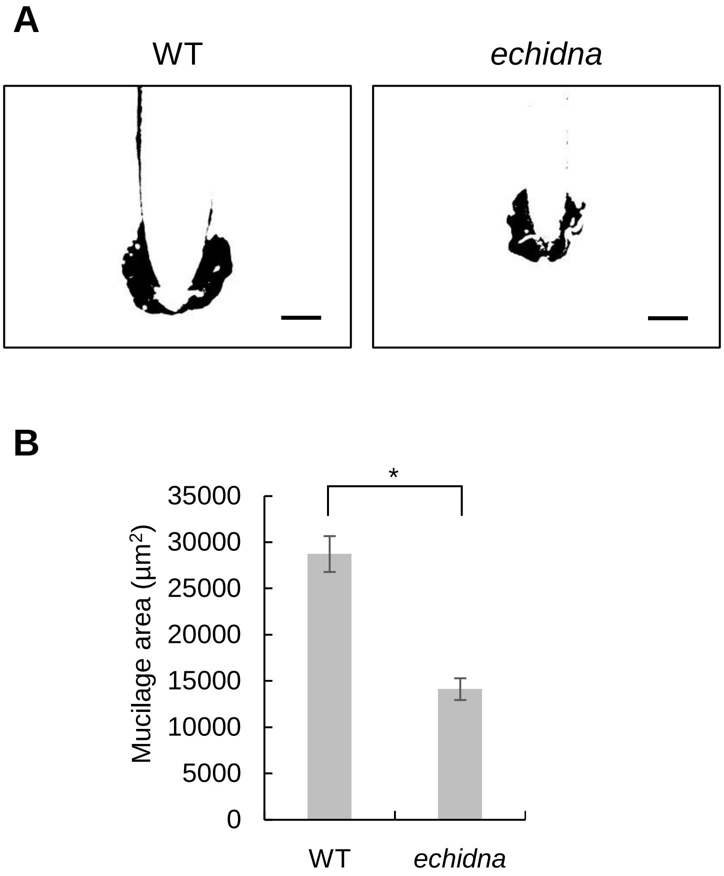

Fig. S5 Representative images converted by Trainable Weka segmentation and quantitative analysis of india ink staining. (A) Distinguished images of root tip of wild-type (WT) and *echidna* mutant stained with india ink (Fig. 4C). Bars, 50 µm. (B) Quantitative analysis of mucilage area of WT and *echidna* mutant. Data represent the mean with standard error. Asterisk indicates statistical significance between WT and *echidna* mutant (p < 0.005) corresponding to Student's t-test. The 32 root tips were measured for each genotype.

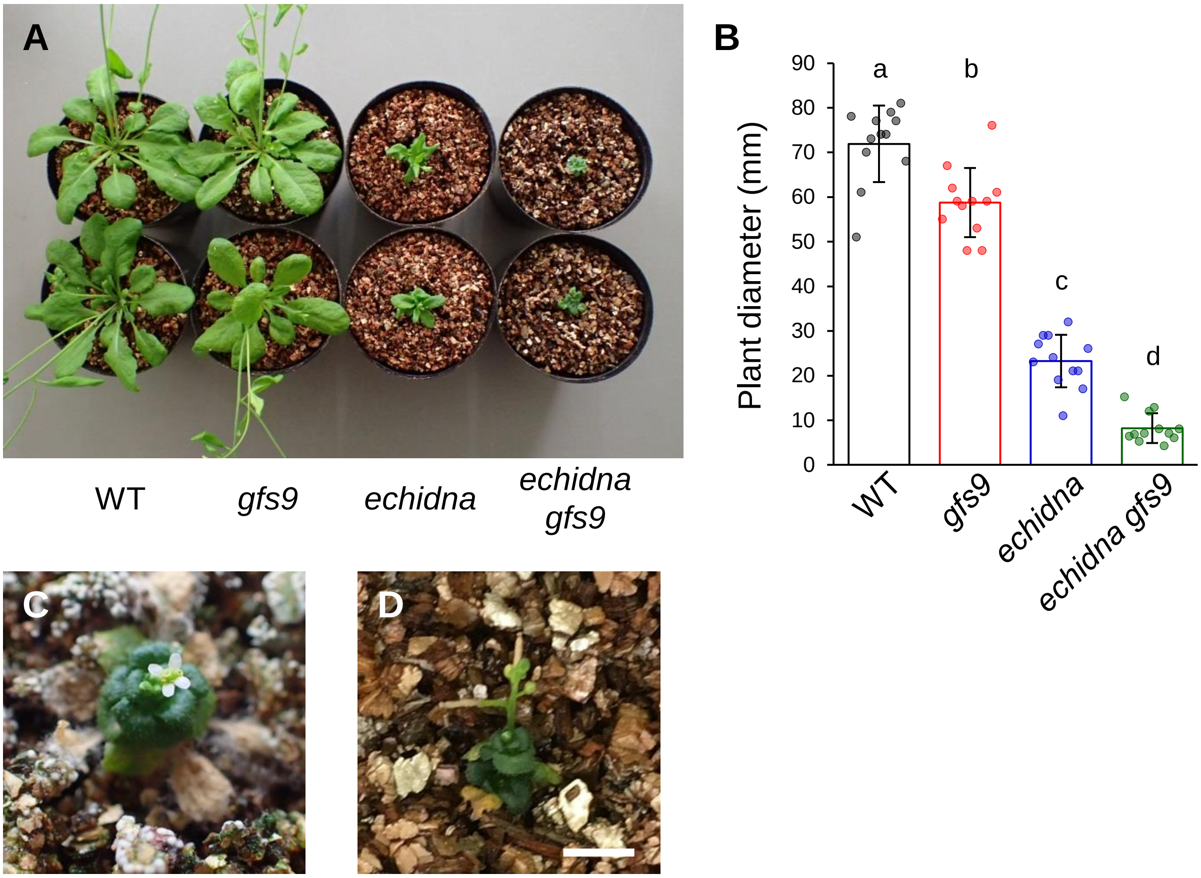

Fig. S6 Plant size and reproductive growth of *echidna gfs9* double mutant. (A, B) The 40-day-old plants (A) and their diameters (B) of wild-type (WT), *gfs9*, *echidna*, and *echidna gfs9* double mutants are shown. The 12 individuals are measured for each genotype. Data represent the average with S.D. and raw data points. Letters indicate statistical significance corresponding to Tukey's HSD test for multiple comparisons ($\alpha = 0.01$). (C, D) The 69-day-old (C) and 80-day-old (D) *echidna gfs9* double mutants are shown to be fairly infrequently successful in bolting and flowering. Bar, 5 mm.
